# *Acinetobacter calcoaceticus* CSY-P13 Mitigates Stress of Ferulic and *p*-Hydroxybenzoic Acids in Cucumber by Affecting Antioxidant Enzyme Activity and Soil Bacterial Community

**DOI:** 10.3389/fmicb.2018.01262

**Published:** 2018-06-14

**Authors:** Fenghui Wu, Yan-Qiu An, Yanrong An, Xiu-Juan Wang, Zeng-Yan Cheng, Yue Zhang, Xinwei Hou, Chang-Xia Chen, Li Wang, Ji-Gang Bai

**Affiliations:** ^1^State Key Laboratory of Crop Biology, College of Life Sciences, Shandong Agricultural University, Tai’an, China; ^2^State Key Laboratory of Crop Biology, College of Horticulture Science and Engineering, Shandong Agricultural University, Tai’an, China

**Keywords:** *Acinetobacter calcoaceticus*, antioxidant enzyme, bacterial community, cucumber, ferulic acid, *p*-hydroxybenzoic acid

## Abstract

Ferulic acid (FA) and *p*-hydroxybenzoic acid (PHBA) are main phenolic compounds accumulated in rhizosphere of continuously cropped cucumber, causing stress in plants. Microbial degradation of a mixture of FA and PHBA is not well understood in soil. We isolated a strain CSY-P13 of *Acinetobacter calcoaceticus*, inoculated it into soil to protect cucumber from FA and PHBA stress, and explored a mechanism underlying the protection. CSY-P13 effectively degraded a mixture of FA and PHBA in culture solution under conditions of 39.37°C, pH 6.97, and 21.59 g L^-1^ potassium dihydrogen phosphate, giving rise to 4-vinyl guaiacol, vanillin, vanillic acid, and protocatechuic acid. During FA and PHBA degradation, activities of superoxide dismutase (SOD), catalase, ascorbate peroxidase, and dehydroascorbate reductase in CSY-P13 were induced. Inoculated into cucumber-planted soil containing 220 μg g^-1^ mixture of FA and PHBA, CSY-P13 degraded FA and PHBA in soil, increased plant height, and decreased malonaldehyde, superoxide radical, and hydrogen peroxide levels in leaves. CSY-P13 also enhanced SOD, guaiacol peroxidase, catalase, glutathione peroxidase, ascorbate peroxidase, monodehydroascorbate reductase, dehydroascorbate reductase, and glutathione reductase activities; increased ascorbate and glutathione contents; and elevated transcript levels of copper/zinc SOD, manganese SOD, and catalase in leaves under FA and PHBA. Moreover, CSY-P13 increased phosphatase, catalase, urease, and sucrase activities and changed bacterial richness, diversity, and community composition by high throughput sequencing in cucumber-planted soil supplemented with the mixture of FA and PHBA. So CSY-P13 degrades the mixture of FA and PHBA in soil and mitigates stress from the two phenolic compounds in cucumber by activating antioxidant enzymes, changing soil bacterial community, and inducing soil enzymes.

## Introduction

Many crops including cucumber are often grown repeatedly in a same soil in intensive agriculture. As a result, phenolic compounds secreted by crop roots are accumulated in the soil. The accumulation of these phenolic compounds has allelopathic stress effects and can inhibit plant growth and development, thereby restraining sustainable crop production (Yu, 2001). When cucumber plants have been continuously cropped for 7 years, ferulic acid (FA) and *p*-hydroxybenzoic acid (PHBA, 4-hydroxybenzoic acid) are the main phenolic compounds, which are accumulated in rhizospheric soil (Xu S.X. et al., 2008). Therefore, degrading a mixture of FA and PHBA in soil may mitigate the allelopathic stress effects of accumulated phenolic compounds on crops such as cucumber.

Microbial degradation of phenolic compounds (Yu, 2001) is cost-effective and environmentally friendly (De et al., 2006). Strain 11 of *Acinetobacter calcoaceticus* is able to utilize a phenolic mixture of PHBA, 2-hydroxybenzoic acid, and 3-hydroxybenzoic acid in medium (Prathibha and Sumathi, 2008). *A. calcoaceticus* DSM586 can grow on PHBA as sole carbon source and also has a capacity of degrading FA in medium (Delneri et al., 1995). Using *Acinetobacter* sp. PK1, phenolic compounds including FA and PHBA are removed from palm oil mill effluent (Khongkhaem et al., 2016). To the best of our knowledge, it has not been reported that strains of *Acinetobacter* might degrade a mixture of FA and PHBA in soil.

To deplete a mixture of FA and PHBA in soil and thereby to protect plants from the stress of the mixed phenolic compounds, a strain CSY-P13 was isolated from rhizospheric soil of continuously cropped cucumber and identified as *A. calcoaceticus* in this study. Then, we applied the strain CSY-P13 to cucumber-planted soil supplemented with a mixture of FA and PHBA. It was hypothesized that *Acinetobacter* strains obtained from continuous cropping soil might have a phenolic compound-degrading ability, thus decomposing a mixture of FA and PHBA in soil and protecting plants under phenolic compound-contained conditions.

We next explored a mechanism underlying the protection effects of CSY-P13. Addition of FA (Zhang et al., 2015) or PHBA (Chen et al., 2015) to cucumber-planted soil causes oxidative stress in seedlings, which leads to overproduction of reactive oxygen species (ROS) including superoxide radical (${\text{O}}_{2}^{•-}$) and hydrogen peroxide (H_2_O_2_). The overproduced ROS inhibits plant growth and even results in cell death (Wan et al., 2015). To control ROS levels, plants evolve an antioxidant system, in which superoxide dismutase (SOD, EC 1.15.1.1), guaiacol peroxidase (GPX, EC 1.11.1.7), catalase (CAT, EC 1.11.1.6), glutathione peroxidase (GSH-Px, EC 1.11.1.9), ascorbate peroxidase (APX, EC 1.11.1.11), monodehydroascorbate reductase (MDHAR, EC 1.6.5.4), dehydroascorbate reductase (DHAR, EC 1.8.5.1), and glutathione reductase (GR, EC 1.6.4.2) are common antioxidant enzymes, and ascorbate (AsA) and reduced glutathione (GSH) are important antioxidants (Zhang et al., 2012). SOD dismutates ${\text{O}}_{2}^{•-}$ into H_2_O_2_ and O_2_, and GPX, CAT, GSH-Px, and APX regulate H_2_O_2_ levels, while GSH-dependent DHAR and GR (an enzyme for GSH regeneration) and/or MDHAR regenerate AsA to act as a substrate for APX (Wan et al., 2015). In addition, phenolic compounds influence soil enzyme activities (Chen et al., 2015; Zhang et al., 2015) and change microbial communities in cucumber-planted soil (Zhou et al., 2012). Therefore, we determined antioxidant enzyme activities in cucumber, analyzed soil enzyme activities, and detected microbial communities in soil. Our hypothesis was that CSY-P13 degraded a mixture of FA and PHBA in cucumber-planted soil, induced antioxidant enzymes in seedlings, activated soil enzymes, and changed microbial communities in soil, mitigating the stress of FA and PHBA in cucumber. Moreover, stress-responsive proteins in a phenol-degrading *Acinetobacter* strain contribute to the tolerance of this microorganism to phenol stress (Lin, 2017). During PHBA degradation, oxidative stress-responsive antioxidant enzymes are induced in a PHBA-degrading *Pseudomonas* due to exposure of this bacterium to PHBA (Chen et al., 2015). Thus, when CSY-P13 was used to degrade a mixture of FA and PHBA in this study, the activities of antioxidant enzymes in this strain were analyzed to investigate a degradation mechanism of CSY-P13.

## Materials and Methods

### Isolation, Identification, and Growth Conditions of Strain CSY-P13

When cucumber has been continuously cropped for 3 years, a PHBA-degrading strain CSY-P13 was isolated from rhizosphere soil in late autumn of the third year by using M-9 medium (Turner and Rice, 1975) supplemented with 0.5 g L^-1^ PHBA according to Chen et al. (2015). To analyze the FA-degrading ability of CSY-P13, this strain was then inoculated into M-9 medium containing 0.5 g L^-1^ FA (Zhang et al., 2015). After that, a fragment that targets 10 variable regions in the 16S rRNA gene was amplified from CSY-P13 with the universal primers 1492R and 27F (Wang et al., 2011), and its sequence was deposited in GenBank under an accession number MG773121. By using the 16S rRNA gene sequence, sequence alignment was conducted with the database of National Center for Biotechnology Information (NCBI), and phylogenetic analysis was performed with the Molecular Evolutionary Genetics Analysis (MEGA) 7.0 software package. Then, we analyzed the physiological and biochemical properties of CSY-P13, including nitrate reduction; indole production (Pagel and Seyfried, 1976); Gram staining (Buck, 1982); gelatin liquefaction; glucose fermentation (Bouvet and Grimont, 1986); motility; sucrase activity; utilization of citrate, mannitol, and glucose (Kim et al., 2008); and activities of catalase and oxidase (Lee and Lee, 2010). The strain CSY-P13 was identified based on the sequence alignment and phylogeny analysis of the 16S rRNA gene sequence, combined with the physiological and biochemical properties with reference to the Bergey’s Manual of Determinative Bacteriology.

To incubate CSY-P13, the strain was cultured in Luria-Bertani (LB) liquid medium (containing 10 g L^-1^ tryptone, 5 g L^-1^ yeast extract, and 10 g L^-1^ NaCl) at 28°C and 180 rpm. Then, 1 mL of exponentially growing culture was inoculated into 100 mL of M-9 liquid medium in a 250-mL Erlenmeyer flask. Cell growth of CSY-P13 was measured by the optical density of culture solutions at 600 nm (OD_600_). The FA to PHBA ratio was 57.18: 42.82 (w/w) in a mixture of FA and PHBA in this study, with reference to the concentrations of FA (16.56 μg g^-1^ soil) and PHBA (12.40 μg g^-1^ soil) in rhizosphere soil, where cucumber has been continuously cropped for 7 years (Xu S.X. et al., 2008).

### Concentrations of FA and PHBA in Culture Solutions and Soil

The concentrations of FA and PHBA in culture solutions and soil were determined using high performance liquid chromatography (HPLC) with an Agilent 1200 Rapid Resolution Liquid Chromatography system (Agilent Technologies, Germany) according to Chen et al. (2015) and Zhang et al. (2015).

### Properties of CSY-P13

The properties of CSY-P13 were investigated by analyzing the percentages of degraded FA and PHBA in culture solutions with HPLC. To examine the optimum initial concentration of the mixture of FA and PHBA in the ratio of 57.18: 42.82, CSY-P13 was inoculated into M-9 medium supplemented with 0.1, 0.2, 0.3, 0.4, or 0.5 g L^-1^ mixture of FA and PHBA, and then incubated at 28°C and 180 rpm for 6 h. To detect the optimum nitrogen source, CSY-P13 was inoculated into M-9 medium, in which 0.2 g L^-1^ mixture of FA and PHBA (including 114.4 mg L^-1^ FA and 85.6 mg L^-1^ PHBA) was supplemented, and 1 g L^-1^ NH_4_C1 was changed into the same molar mass of nitrogen from (NH_4_)_2_SO_4_, NaNO_3_, NH_4_NO_3_, or urea. Subsequently, the strain was incubated at 28°C and 180 rpm for 6 h. To identify the optimum culture temperature, CSY-P13 was inoculated into M-9 medium supplemented with 0.2 g L^-1^ mixture of FA and PHBA, and incubated for 3 h at 180 rpm and different temperatures (18, 23, 28, 37, 39, 40, 41, 43, or 45°C). To determine the optimum shaking speed, CSY-P13 was inoculated into M-9 medium supplemented with 0.2 g L^-1^ mixture of FA and PHBA, and incubated for 2.5 h at 40°C with shaking at different speeds (130, 160, 180, 200, or 230 rpm). To measure the optimum pH for CSY-P13, M-9 medium was supplemented with 0.2 g L^-1^ mixture of FA and PHBA, and its initial pH value was adjusted to different values (4, 5, 6, 7, or 8) with 1 M HCl or NaOH. Then, CSY-P13 was inoculated into this medium and incubated at 40°C and 180 rpm for 2 h. To analyze the optimum initial concentration of KH_2_PO_4_, CSY-P13 was inoculated into M-9 medium, in which 0.2 g L^-1^ mixture of FA and PHBA was supplemented, and the initial concentrations of KH_2_PO_4_ were changed into 10, 20, 30, 40, or 50 g L^-1^. After that, the strain was incubated at 40°C with shaking (180 rpm) for 2 h.

### Response Surface Optimization for FA and PHBA Degradation by CSY-P13

On the basis of the properties of CSY-P13, KH_2_PO_4_ concentration, culture temperature, and pH were used to optimize the degradation of the mixture of FA and PHBA by Box–Behnken design. The experimental design with the levels and codes of independent variables is given in Supplementary Table S1, and the predicted percentages of degraded FA and PHBA are shown in Supplementary Table S2.

### Antioxidant Enzyme Activities in CSY-P13

CSY-P13 was inoculated into M-9 medium supplemented with 0.2 g L^-1^ mixture of FA and PHBA and incubated for 2 h under conditions optimized by the response surface methodology. As a control, CSY-P13 was inoculated into M-9 medium supplemented with 306.88 mg L^-1^ glucose, in which the numbers of carbon atoms were equivalent to those in 0.2 g L^-1^ mixture of FA and PHBA. Since 0.2 g L^-1^ mixture of FA and PHBA included 114.4 mg L^-1^ FA and 85.6 mg L^-1^ PHBA, the strain CSY-P13 was also inoculated into M-9 medium supplemented with 114.4 mg L^-1^ FA or 85.6 mg L^-1^ PHBA as another two controls. When culture solutions were centrifuged at 4°C and 5000 *g* for 20 min, cell pellets were collected and ground in liquid nitrogen. Then, cell powder (0.1 g) was soaked into 1 mL of 100 mM potassium phosphate buffer (pH 7.5) containing 1 mM ethylenediaminetetraacetic acid (EDTA), 3 mM DL-dithiothreitol, and 5% (w/v) polyvinylpolypyrrolidone (PVP) (Gratão et al., 2008). After centrifugation at 4°C and 10,000 *g* for 20 min, the resulting supernatants were used to assay the activities of SOD, APX, CAT, and DHAR due to the reduction of nitroblue tetrazolium (NBT), oxidation of AsA, decompounding of H_2_O_2_, and formation of AsA, respectively (Zhang et al., 2012). One unit of SOD activity was defined as the amount of enzyme that gave 50% inhibition of NBT reduction. One unit of CAT, APX, and DHAR activity was defined as 1 μmol of H_2_O_2_ consumed per minute, 1 μmol of AsA oxidized in one minute, and 1 nmol of AsA formed per minute, respectively.

### Degradation Products of FA and PHBA by CSY-P13

CSY-P13 was inoculated into M-9 medium supplemented with 0.2 g L^-1^ mixture of FA and PHBA, 114.4 mg L^-1^ FA, or 85.6 mg L^-1^ PHBA, respectively. Then, the strain was incubated for 2 h under conditions optimized by the response surface methodology. The concentrations of degradation products from FA and PHBA by CSY-P13 were determined with HPLC according to Chen et al. (2015) and Zhang et al. (2015).

### Degradation of the Mixture of FA and PHBA in Soil

In order to grow cucumber seedlings, soil with the following characteristics was autoclaved at 121°C for 20 min and then air-dried at 30°C until a constant weight was reached: pH 7.34, 1.37 g kg^-1^ total N, 29.95 mg kg^-1^ NO_3_-N, 13.87 mg kg^-1^ NH_4_-N, 20.96 mg kg^-1^ Olsen-P, 86.76 mg kg^-1^ rapidly available K, 157.7 μs cm^-1^ electrical conductivity, and 0.75% organic matter. To apply CSY-P13 to soil, the strain was cultured in 100 mL of LB liquid medium at 28°C until OD_600_ = 1. Then, the culture solutions were centrifuged at 4°C and 5000 *g* for 20 min. The resulting cell pellets were resuspended into autoclaved water to reach a concentration of 5.84 log cfu mL^-1^.

Each seed of cucumber (*Cucumis sativus* cv. Jinchun no. 4) was germinated at 25°C for 2 days, and then transferred to a 10-cm-diameter plastic pot containing 450 g of autoclaved soil. Having been grown at 25°C with a photoperiod of 12 h light (600 μmol m^-2^ s^-1^)/12 h dark until the two-leaf stage, 32 cucumber seedlings were chosen and divided into four groups (eight plants per group) to impose the following treatments: I (control group), watered with 25 mL of autoclaved water; II (FA+PHBA treatment group), watered with 25 mL of solutions (containing 5.03 g L^-1^ FA and 3.77 g L^-1^ PHBA) to make the mixture of FA and PHBA reach a concentration of 220 μg g^-1^ dry soil, which induced stress but did not cause plant wilting; III (CSY-P13 treatment group), watered with 25 mL of 5.84 log cfu mL^-1^ CSY-P13 to reach a concentration of 7.24 log cfu plant^-1^; IV (CSY-P13+FA+PHBA treatment group), watered with 25 mL of solutions (containing 5.84 log cfu mL^-1^ CSY-P13, 5.03 g L^-1^ FA, and 3.77 g L^-1^ PHBA) to make CSY-P13 and the mixture of FA and PHBA reach the concentrations of 7.24 log cfu plant^-1^ and 220 μg g^-1^ dry soil, respectively. After that, the seedlings were maintained at 40% (w/w) soil moisture with autoclaved H_2_O under conditions of 25°C and 12 h light (600 μmol m^-2^ s^-1^)/12 h dark. At 20 days after inoculation with CSY-P13, the fully expanded second leaves and rhizospheric soil of cucumber were collected. This experiment was repeated three times.

### Levels of Malondialdehyde, ${\text{O}}_{2}^{•-}$, and H_2_O_2_ in Leaves

Malondialdehyde (MDA) in cucumber leaves was extracted with 10% trichloroacetic acid, and its content was determined by thiobarbituric acid assay at 450, 532, and 600 nm (Xu P.L. et al., 2008). The formation rate of ${\text{O}}_{2}^{•-}$ was determined at 530 nm according to Elstner and Heupel (1976). The content of H_2_O_2_ was measured by monitoring a titanium peroxide complex at 415 nm (Mukherjee and Choudhuri, 1983).

### Activities of Antioxidant Enzymes in Leaves

Leaf sample (0.1 g) was ground with liquid nitrogen and suspended in 1 mL of HEPES buffer (25 mM, pH 7.8) containing 0.2 mM EDTA and 2% (w/v) PVP. After centrifugation at 4°C and 12,000 *g* for 20 min, the supernatant was the extract of SOD, CAT, GSH-Px, MDHAR, DHAR, and GR. To extract APX, 0.02 g of liquid nitrogen-ground cucumber leaves was suspended in 3 mL of HEPES buffer (25mM, pH 7.8), which contained 0.2 mM EDTA, 2% (w/v) PVP, and 2 mM AsA. The activities of SOD, CAT, APX, and DHAR were assayed according to Zhang et al. (2012) as indicated above. The activities of MDHAR, GSH-Px, and GR were measured due to the oxidation of NADH, decrease in GSH, and oxidation of NADPH, respectively (Zhang et al., 2012). One unit of GSH-Px, MDHAR, and GR activity was defined as 1 μmol of GSH decreased per minute, 1 nmol of NADH oxidized in one minute, and 1 μmol of NADPH oxidized per minute, respectively.

### Transcript Levels of *Cu/Zn-SOD*, *Mn-SOD*, and *CAT* in Leaves

To confirm the activities of SOD and CAT, transcript levels of *Cu/Zn-SOD*, *Mn-SOD*, and *CAT* in cucumber leaves were estimated using real-time quantitative RT-PCR. Total RNA was extracted from cucumber leaves with a TRIzol reagent (Invitrogen, Carlsbad, United States) and reverse-transcribed to cDNA using a Quantscript RT kit (Cwbio, China). PCR primers of *Cu/Zn-SOD*, *Mn-SOD*, and *CAT* are listed in Supplementary Table S3. The PCR reaction mixture (20 μL) contained 10 μL of 2× ultra SYBR Mixture (with Rox; Cwbio, China), 2 μL of template cDNA, and 0.8 μL of 5 pM forward and reverse primers. PCR parameters included an initial denaturation step at 95°C for 10 min, followed by 40 amplification cycles of 15 s at 95°C, and 1 min at 55°C. PCR amplification was performed with the CFX96^TM^ real-time system (Bio-Rad, Hercules, United States) in triplicate. The transcript levels of *Cu/Zn-SOD*, *Mn-SOD*, and *CAT* were normalized against the corresponding *actin* mRNA levels, and calculated with a 2^-ΔΔCt^ comparative CT method.

### Antioxidant Contents in Leaves

To verify the activities of DHAR, MDHAR, and GR, the level of AsA was determined at 525 nm in cucumber leaves according to Klein and Perry (1982), and the content of GSH was measured at 412 nm as described by Guri (1983).

### Activities of Soil Enzymes in Rhizospheric Soil

The activities of CAT (Roberge, 1978), sucrase (Schinner and Von Mersi, 1990), urease (Douglas and Bremner, 1971), and alkaline phosphatase (Anupama et al., 2008) were assayed in cucumber rhizospheric soil due to the decompounding of H_2_O_2_, formation of reducing sugars from sucrose, production of NH_3_-N from urea, and formation of phosphate from disodium phenyl phosphate, respectively. One unit of CAT, sucrase, urease, and alkaline phosphatase activity was, respectively, defined as 1 mL of KMnO_4_ consumed in 20 min, 1 mg of reducing sugars formed in 24 h, 1 mg of NH_3_-N produced in 24 h, and 1 mg of P_2_O_5_ produced in 2 h.

### DNA Extraction, High Throughput Sequencing, and Data Processing of Soil Microorganisms in Rhizospheric Soil

Soil microorganism DNA was extracted from cucumber rhizosphere soil using an EZNA^®^ Soil DNA Kit (Omega Bio-tek, Norcross, GA, United States). Then, high throughput sequencing and data processing for 16S rRNA genes were performed at Shanghai Personal Biotechnology Co., Ltd. according to the procedures of Tao et al. (2017). Raw sequences data for bacterial 16S rRNA genes were deposited in NCBI’s Sequence Read Archive (SRA) with the accession number SRP128603. Relative abundance of an operational taxonomic unit (OTU) was given by the number of clone sequences in each OTU in relation to the total number of clones in each sample.

### Statistical Analysis

All data are presented as means ± standard errors of three biological replicates. One-way analysis of variance (ANOVA) and the least significant difference (LSD) were performed to analyze differences among treatments using SPSS for Windows 22.0 software (IBM Corp., Armonk, NY, United States). *P*-Values less than 0.05 were considered significant.

## Results

### Identification of CSY-P13

Phylogenetic analysis (Supplementary Figure S1) and sequence alignment of 16S rRNA gene sequences showed that CSY-P13 exhibited 100% identity with *A. calcoaceticus.* Also, CSY-P13 showed the physiological and biochemical characteristics of *Acinetobacter* (Supplementary Table S4). Thus, this isolate was identified as *A. calcoaceticus* CSY-P13.

### Properties of CSY-P13

When CSY-P13 was inoculated for 6 h, the percentages of degraded FA and PHBA decreased with increasing initial concentrations of the mixture of FA and PHBA (Supplementary Figure S2A). CSY-P13 degraded FA and PHBA completely in 0.1 g L^-1^ mixture of FA and PHBA. However, the strain decomposed 91.28% FA and 87.26% PHBA in 0.2 g L^-1^ mixture of FA and PHBA and resulted in the highest amounts of degraded FA and PHBA and the best cell growth. Therefore, 0.2 g L^-1^ was chosen as the optimum initial concentration of the mixture of FA and PHBA.

As NH_4_Cl in M-9 medium was replaced by ammonium sulfate, sodium nitrate, and urea, the percentages of degraded FA or PHBA by CSY-P13 did not change significantly (*P* > 0.05) (Supplementary Figure S2B). Under condition of NH_4_Cl, cell growth of CSY-P13 was the best (*P* < 0.05). So NH_4_Cl was the optimum nitrogen source.

The percentages of degraded FA and PHBA by CSY-P13 both increased from 18 to 40°C and reached a maximum at 40°C (Supplementary Figure S2C). From 40 to 45°C, the percentages of degraded FA and PHBA decreased. Thus, 40°C was the optimum culture temperature of CSY-P13.

The percentages of degraded FA and PHBA increased from 130 to 180 rpm, and then decreased from 180 to 230 rpm (Supplementary Figure S2D). Incubating CSY-P13 at a shaking speed of 180 rpm for 2.5 h, the percentages of degraded FA and PHBA reached a maximum. Therefore, 180 rpm was chosen as the optimum shaking speed of CSY-P13.

At pH 7, the percentage of degraded FA by CSY-P13 was higher (*P* < 0.01) than that at pH 6 or 8 (Supplementary Figure S2E). After CSY-P13 inoculation, the percentage of degraded PHBA increased from pH 4 to 6 and decreased from pH 6 to 8. At pH 6, the percentage of degraded PHBA by CSY-P13 had no change (*P* > 0.05) in comparison to that at pH 7. Thus, pH 7 was the optimum pH for FA and PHBA degradation by CSY-P13.

Incubating CSY-P13 in 10, 20, 30, 40, or 50 g L^-1^ KH_2_PO_4_ for 2 h, the percentages of degraded FA and PHBA and cell growth all increased from 10 to 30 g L^-1^ KH_2_PO_4_ and then decreased from 30 to 50 g L^-1^ KH_2_PO_4_ (Supplementary Figure S2F). With 30 g L^-1^ KH_2_PO_4_, the percentages of degraded FA and PHBA by CSY-P13 reached maximum amounts. Therefore, 30 g L^-1^ was the optimum concentration of KH_2_PO_4_.

### Response Surface Optimization for FA and PHBA Degradation by CSY-P13

A polynomial model was performed by using multiple regression analysis to show an interaction between variables in coded units and factors that influenced FA and PHBA degradation by CSY-P13. Coefficient estimates of the model and their ANOVA results (Supplementary Tables S5, S6) indicated that the KH_2_PO_4_ concentration, culture temperature, and pH were correlated with the percentages of degraded FA and PHBA by CSY-P13. As shown in a 3D response surface (Supplementary Figures S3, S4), optimal values of the variables were temperature 39.37°C, pH 6.97, and KH_2_PO_4_ concentration of 21.59 g L^-1^. Incubating CSY-P13 for 2 h under the predicted optimal conditions, the percentages of degraded FA and PHBA were 74.29 and 72.36%, respectively, which were in agreement with the predicted degradation rates of FA (74.45%) and PHBA (72.26%) and indicated that the model was adequate for FA and PHBA degradation by CSY-P13.

### Bioconversion Products of the Mixture of FA and PHBA by CSY-P13 in Culture Solutions

**FIGURE 1:**
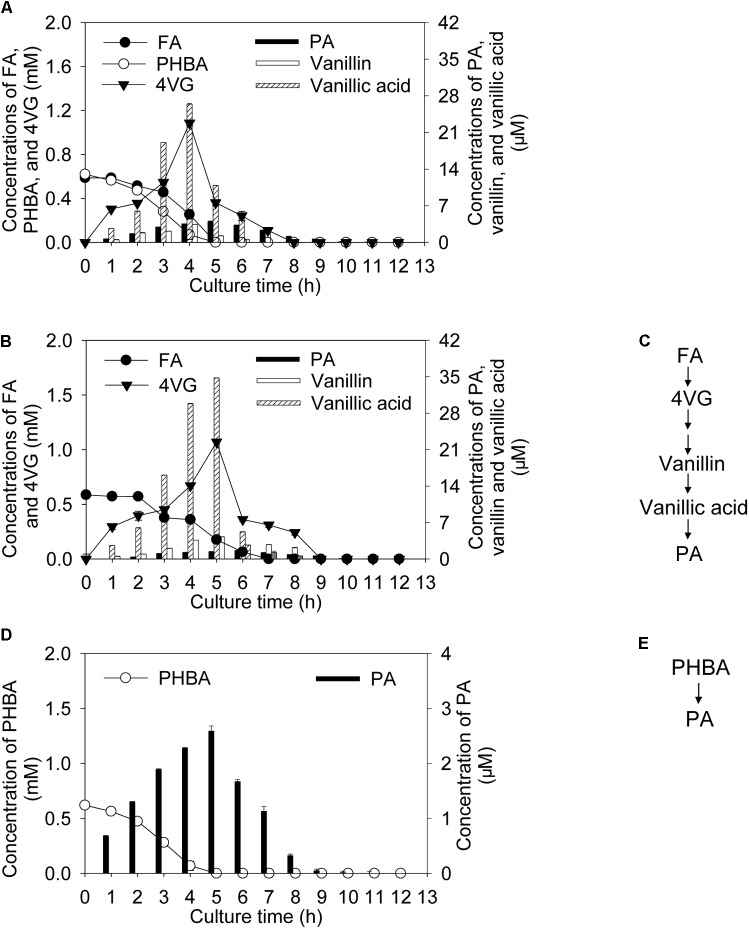
Bioconversion products from the mixture of FA and PHBA **(A)**, FA **(B)**, and PHBA **(D)**, and proposed pathway of FA **(C)** and PHBA **(E)** metabolism by CSY-P13. Bars represent standard errors of three biological replicates.

Incubating CSY-P13 for 2 h under conditions optimized by the response surface methodology, bioconversion products of the mixture of FA and PHBA included 4-vinyl guaiacol (4VG), protocatechuic acid (PA), vanillin, and vanillic acid (**Figure 1A**). Among these, the metabolites of FA contained 4VG and small quantities of PA, vanillin, and vanillic acid (**Figures 1B,C**); and only PA was detected during PHBA degradation (**Figures 1D,E**). During the degradation of the mixture of FA and PHBA by CSY-P13, concentrations of 4VG, vanillin, and vanillic acid increased from 0 to 4 h, and they reached a maximum at 4 h and then decreased (**Figure 1A**). Meanwhile, PA reached a maximum yield at 5 h.

### Effects of the Mixture of FA and PHBA on Antioxidant Enzyme Activity in CSY-P13

**FIGURE 2:**
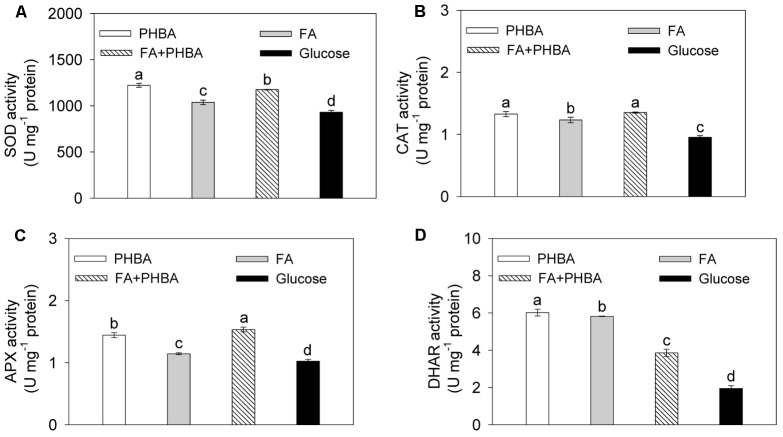
Activities of SOD **(A)**, CAT **(B)**, APX **(C)**, and DHAR **(D)** in CSY-P13. PHBA, cultured in PHBA; FA, cultured in FA; FA+PHBA, cultured in the mixture of FA and PHBA; glucose, cultured in glucose. Bars represent standard errors of three biological replicates. Values with the different letters are significantly different at *P* < 0.05 by LSD test using SPSS for Windows 22.0 software (IBM Corp., Armonk, NY, United States).

Under conditions optimized by the response surface methodology, the activities of SOD (**Figure 2A**), CAT (**Figure 2B**), APX (**Figure 2C**), and DHAR (**Figure 2D**) in CSY-P13 were increased (*P* < 0.05) by the treatment of FA+PHBA or FA alone or PHBA alone compared to the glucose treatment.

### Degradation of the Mixture of FA and PHBA by CSY-P13 in Soil

**FIGURE 3:**
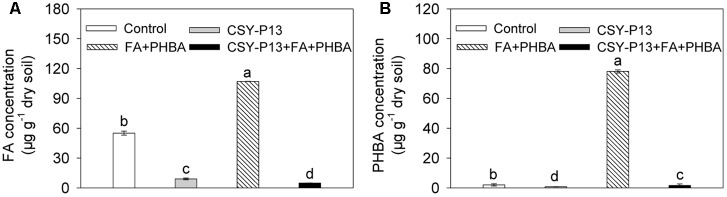
FA **(A)** and PHBA **(B)** degradation by CSY-P13 in cucumber-planted soil. Control, supplemented with water; FA+PHBA, supplemented with the mixture of FA and PHBA; CSY-P13, supplemented with CSY-P13; CSY-P13+FA+PHBA, supplemented with CSY-P13 and the mixture of FA and PHBA. Bars represent standard errors of three biological replicates. Values with the different letters are significantly different at *P* < 0.05 by LSD test using SPSS for Windows 22.0 software (IBM Corp., Armonk, NY, United States).

Compared to control, the concentrations of FA (**Figure 3A**) and PHBA (**Figure 3B**) remaining in soil were decreased by 83.66% (*P* < 0.001) and 63.02% (*P* < 0.001), respectively, in the CSY-P13 treatment. In the CSY-P13+FA+PHBA treatment in comparison to the FA+PHBA treatment, the concentrations of FA and PHBA remaining were decreased by 95.48% (*P* < 0.001) and 97.93% (*P* < 0.001), respectively.

### Effects of CSY-P13 and the Mixture of FA and PHBA on Plant Height

**FIGURE 4:**
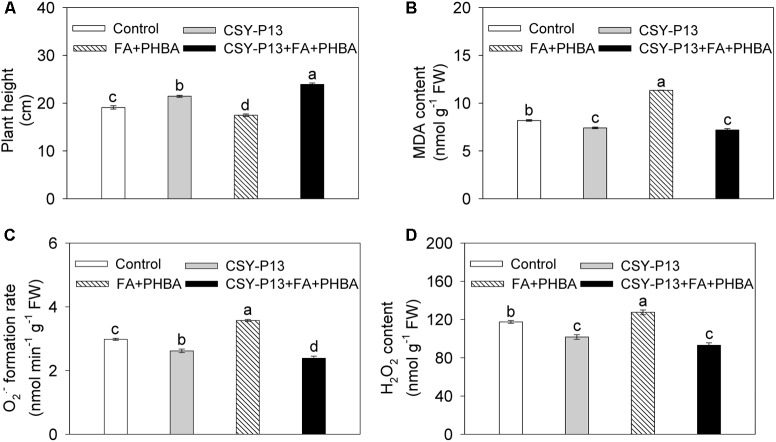
Effects of CSY-P13 and the mixture of FA and PHBA on plant height **(A)**, and levels of MDA **(B)**, ${\text{O}}_{2}^{•-}$**(C)**, and H_2_O_2_ **(D)** in cucumber leaves. Bars represent standard errors of three biological replicates. Values with the different letters are significantly different at *P* < 0.05 by LSD test using SPSS for Windows 22.0 software (IBM Corp., Armonk, NY, United States).

Compared to control, plant height of cucumber was increased by 12.21% (*P* < 0.05) in the CSY-P13 treatment and decreased by 8.55% (*P* < 0.05) in the FA+PHBA treatment (**Figure 4A**). In the CSY-P13+FA+PHBA treatment in comparison to the FA+PHBA treatment, the plant height was increased by 37.02% (*P* < 0.001).

### Effects of CSY-P13 and the Mixture of FA and PHBA on MDA, ${\text{O}}_{2}^{•-}$, and H_2_O_2_ Levels in Leaves

In comparison to control, the levels of MDA (**Figure 4B**), ${\text{O}}_{2}^{•-}$ (**Figure 4C**), and H_2_O_2_ (**Figure 4D**) in cucumber leaves were decreased by 9.49% (*P* < 0.01), 12.15% (*P* < 0.01), and 13.41% (*P* < 0.01), respectively, in the CSY-P13 treatment and increased by 38.52% (*P* < 0.01), 19.88% (*P* < 0.001), and 8.53% (*P* < 0.05), respectively, in the FA+PHBA treatment. Compared the CSY-P13+FA+PHBA treatment to the FA+PHBA treatment, the levels of MDA, ${\text{O}}_{2}^{•-}$, and H_2_O_2_ in cucumber leaves were decreased by 36.65% (*P* < 0.001), 33.18% (*P* < 0.001), and 26.96% (*P* < 0.01), respectively.

### Effects of CSY-P13 and the Mixture of FA and PHBA on Antioxidant Enzyme Activities in Leaves

**FIGURE 5:**
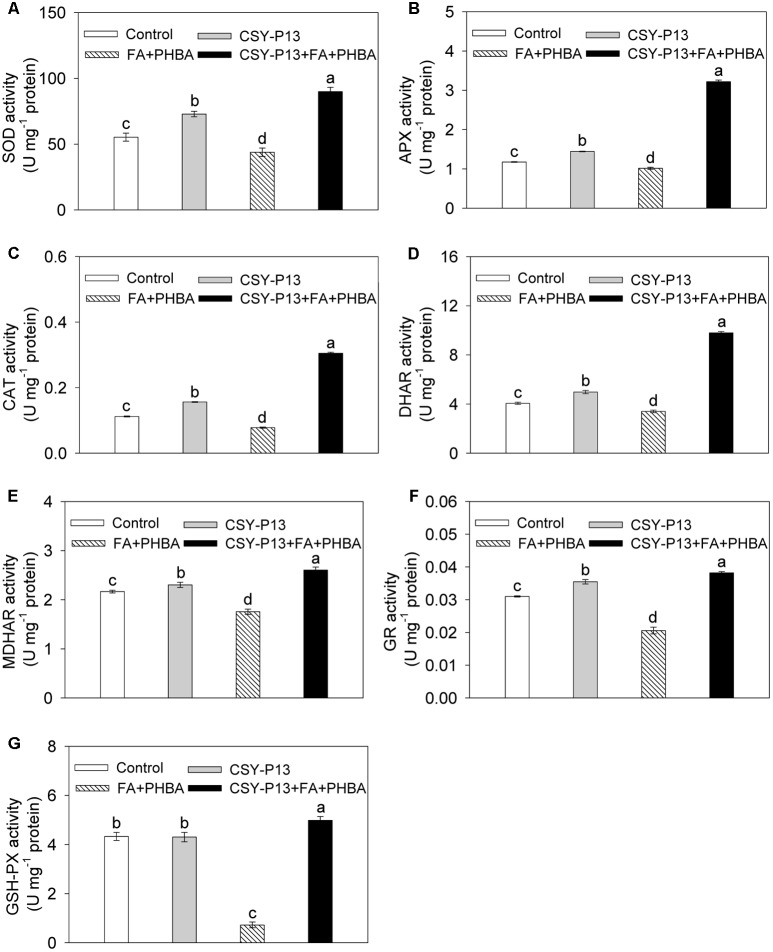
Effects of CSY-P13 and the mixture of FA and PHBA on SOD **(A)**, APX **(B)**, CAT **(C)**, DHAR **(D)**, MDHAR **(E)**, GR **(F)**, and GSH-Px **(G)** activities in cucumber leaves. Bars represent standard errors of three biological replicates. Values with the different letters are significantly different at *P* < 0.05 by LSD test using SPSS for Windows 22.0 software (IBM Corp., Armonk, NY, United States).

Compared to control, the activities of SOD (**Figure 5A**), APX (**Figure 5B**), CAT (**Figure 5C**), DHAR (**Figure 5D**), MDHAR (**Figure 5E**), and GR (**Figure 5F**) in cucumber leaves were, respectively, increased by 31.7% (*P* < 0.001), 22.86% (*P* < 0.001), 39.49% (*P* < 0.001), 22.57% (*P* < 0.001), 6.33% (*P* < 0.05), and 14.51% (*P* < 0.001) in the CSY-P13 treatment, and, respectively, decreased by 20.74% (*P* < 0.001), 12.76% (*P* < 0.01), 30.46% (*P* < 0.001), 16.26% (*P* < 0.001), 18.97% (*P* < 0.001), and 33.56% (*P* < 0.001) in the FA+PHBA treatment. Meanwhile, the activity of GSH-Px (**Figure 5G**) in cucumber leaves did not change (*P* > 0.05) when compared the CSY-P13 treatment with control and decreased by 83.27% (*P* < 0.001) in the FA+PHBA treatment in comparison to control. Compared the CSY-P13+FA+PHBA treatment to the FA+PHBA treatment, the activities of SOD, APX, CAT, GSH-Px, DHAR, MDHAR, and GR in cucumber leaves were enhanced by 51.26% (*P* < 0.001), 68.17% (*P* < 0.001), 74.47% (*P* < 0.001), 84.47% (*P* < 0.001), 65.32% (*P* < 0.001), 32.58% (*P* < 0.001), and 46.1% (*P* < 0.001), respectively.

### Effects of CSY-P13 and the Mixture of FA and PHBA on Transcript Levels of *Cu/Zn-SOD*, *Mn-SOD*, and *CAT* in Leaves

**FIGURE 6:**
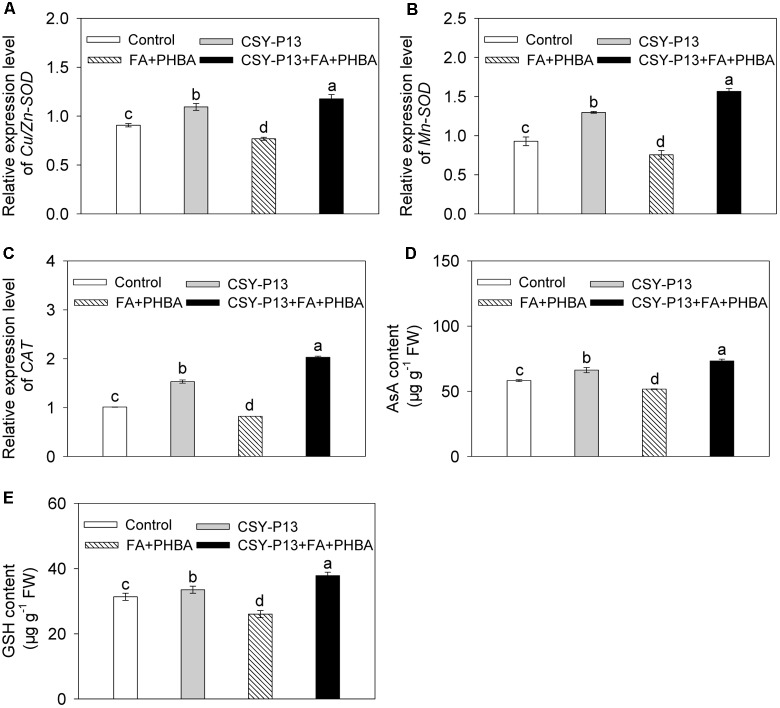
Effects of CSY-P13 and the mixture of FA and PHBA on transcript levels of *Cu/Zn-SOD* **(A)**, *Mn-SOD* **(B)**, and *CAT* **(C)**, and contents of AsA **(D)** and GSH **(E)** in cucumber leaves. Bars represent standard errors of three biological replicates. Values with the different letters are significantly different at *P* < 0.05 by LSD test using SPSS for Windows 22.0 software (IBM Corp., Armonk, NY, United States).

In comparison to control, the transcript levels of *Cu/Zn-SOD* (**Figure 6A**), *Mn-SOD* (**Figure 6B**), and *CAT* (**Figure 6C**) in cucumber leaves were, respectively, increased by 20.67% (*P* < 0.001), 39.9% (*P* < 0.001), and 51.62% (*P* < 0.001) in the CSY-P13 treatment, and, respectively, decreased by 15.27% (*P* < 0.01), 18.64% (*P* < 0.001), and 18.72% (*P* < 0.001) in the FA+PHBA treatment. In the CSY-P13+FA+PHBA treatment in comparison to the FA+PHBA treatment, the transcript levels of *Cu/Zn-SOD*, *Mn-SOD*, and *CAT* in cucumber leaves were increased by 53.13% (*P* < 0.001), 107.45% (*P* < 0.001), and 147.1% (*P* < 0.001), respectively.

### Effects of CSY-P13 and the Mixture of FA and PHBA on AsA and GSH Contents in Leaves

Compared to control, the contents of AsA (**Figure 6D**) and GSH (**Figure 6E**) in cucumber leaves were increased by 13.79% (*P* < 0.01) and 6.88% (*P* < 0.05), respectively, in the CSY-P13 treatment and, respectively, decreased by 11.27% (*P* < 0.05) and 16.96% (*P* < 0.05) in the FA+PHBA treatment. In comparison to the FA+PHBA treatment, the contents of AsA and GSH were increased by 41.92% (*P* < 0.001) and 45.31% (*P* < 0.001), respectively, in cucumber leaves of the CSY-P13+FA+PHBA treatment.

### Effects of CSY-P13 and the Mixture of FA and PHBA on Soil Enzyme Activities

**FIGURE 7:**
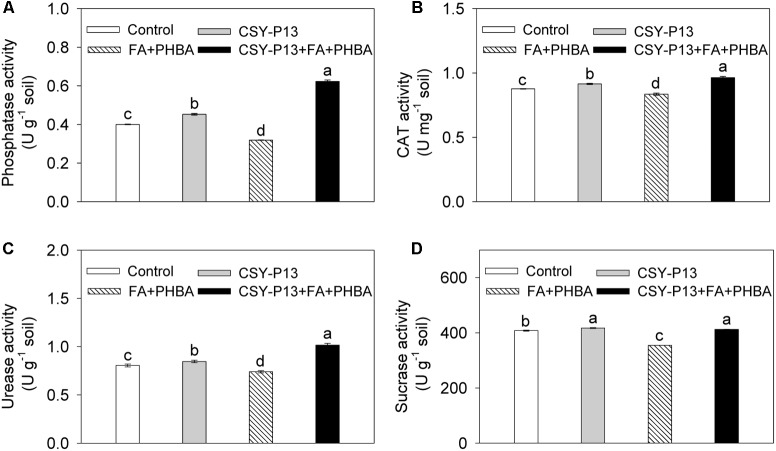
Effects of CSY-P13 and the mixture of FA and PHBA on phosphatase **(A)**, CAT **(B)**, urease **(C)**, and sucrase **(D)** activities in cucumber rhizospheric soil. Bars represent standard errors of three biological replicates. Values with the different letters are significantly different at *P* < 0.05 by LSD test using SPSS for Windows 22.0 software (IBM Corp., Armonk, NY, United States).

In comparison to control, the activities of phosphatase (**Figure 7A**), CAT (**Figure 7B**), urease (**Figure 7C**), and sucrase (**Figure 7D**) in rhizospheric soil were, respectively, increased by 5.23% (*P* < 0.05), 4.45% (*P* < 0.05), 5.07% (*P* < 0.05), and 2.26% (*P* < 0.05) in the CSY-P13 treatment and, respectively, decreased by 20.30% (*P* < 0.001), 4.58% (*P* < 0.05), 8.14% (*P* < 0.05), and 13.13% (*P* < 0.01) in the PHBA+FA treatment. Compared the CSY-P13+FA+PHBA treatment with the FA+PHBA treatment, the activities of phosphatase, CAT, urease, and sucrase in rhizospheric soil were increased by 95.35% (*P* < 0.001), 15.37% (*P* < 0.05), 37.38% (*P* < 0.001), and 16.31% (*P* < 0.001), respectively.

### Effects of CSY-P13 and the Mixture of FA and PHBA on Bacterial Richness and Diversity in Rhizospheric Soil

When low-quality sequences with an average quality score less than 20, length shorter than 150 bp, any mismatches to the primers, or a homopolymer longer than eight bases were removed, 40,498, 43,252, 40,082, and 40,660 high-quality sequences were obtained from the treatments of control, CSY-P13, FA+PHBA, and CSY-P13+FA+PHBA, respectively. All rarefaction curves tended to reach saturation with the increase of sequence numbers (Supplementary Figure S5), suggesting the sufficient sequencing depth was achieved in each treatment.

**FIGURE 8:**
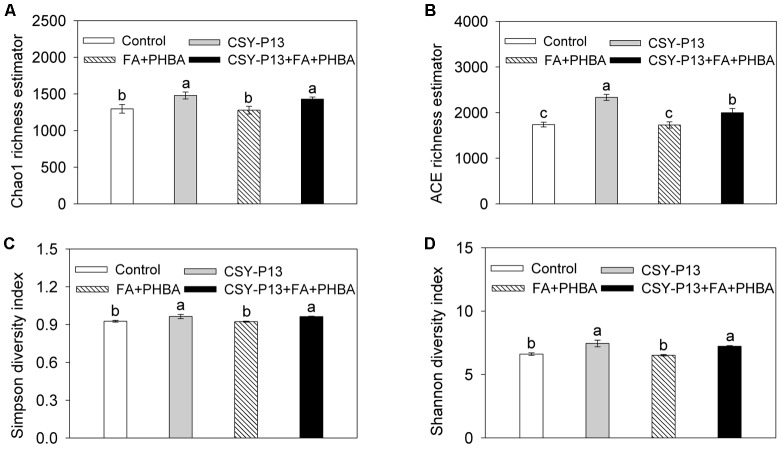
Effects of CSY-P13 and the mixture of FA and PHBA on bacterial richness and diversity in cucumber rhizospheric soil by the Chao1 **(A)** and ACE **(B)** richness estimators and the Simpson **(C)** and Shannon **(D)** indices. Bars represent standard errors of three biological replicates. Values with the different letters are significantly different at *P* < 0.05 by LSD test using SPSS for Windows 22.0 software (IBM Corp., Armonk, NY, United States).

Bacterial richness in rhizospheric soil was evaluated using the Chao1 (**Figure 8A**) and ACE (**Figure 8B**) richness estimators. We examined the effect of CSY-P13, finding that both Chao1 and ACE richness estimators were increased significantly (*P* < 0.05) in the CSY-P13 treatment in comparison to the control or in the CSY-P13+FA+PHBA treatment when compared to the FA+PHBA treatment. So CSY-P13 increased bacterial richness in rhizospheric soil. We next tested the effect of the mixture of FA and PHBA and found that the Chao1 and ACE richness estimators both did not change significantly (*P* > 0.05) between the FA+PHBA treatment and control. This indicated that the mixture of FA and PHBA did not affect bacterial richness in rhizospheric soil.

To investigate bacterial diversity in rhizospheric soil, the Simpson (**Figure 8C**) and Shannon (**Figure 8D**) indices were analyzed. In the CSY-P13 treatment in comparison to the control or in the CSY-P13+FA+PHBA treatment compared to the FA+PHBA treatment, the Simpson and Shannon indices were both increased significantly (*P* < 0.05). Compared the FA+PHBA treatment with control, the Simpson and Shannon indices did not differ (*P* > 0.05). Therefore, CSY-P13 increased bacterial diversity in rhizospheric soil, and the mixture of FA and PHBA did not affect it.

Variations of OTUs were observed among treatments. The number of OTUs in the control, CSY-P13, FA+PHBA, and CSY-P13+FA+PHBA treatments was 3726, 3866, 3620, and 3845, respectively. Venn diagrams showed that bacterial communities of four treatments shared many OTUs (Supplementary Figure S6). There were 2219 shared OTUs detected in the rhizospheric soil of all four treatments. Considerable overlap was observed in the CSY-P13-treated rhizospheric soils, with the CSY-P13 treatment and the CSY-P13+FA+PHBA treatment having 3244 shared OTUs. Similarly, the rhizospheric soils treated with the mixture of FA and PHBA had considerable overlap, with the CSY-P13+FA+PHBA treatment and the FA+PHBA treatment having 3105 shared OTUs.

### Effects of CSY-P13 and the Mixture of FA and PHBA on Bacterial Community Composition and Structure in Rhizospheric Soil

In rhizospheric soil of all four treatments, there were 27 phyla (Supplementary Figure S7A), 63 classes (Supplementary Figure S7B), 103 orders (Supplementary Figure S8A), 184 families (Supplementary Figure S8B), and 389 genera (Supplementary Figure S9). The dominant phyla were Firmicutes, Proteobacteria, Actinobacteria, and Bacteroidetes, respectively, representing 50.17, 29.76, 11.44, and 5.00% in the rhizospheric soil of control; 51.34, 33.07, 7.84, and 4.57% in the CSY-P13 treatment; 47.98, 39.84, 4.21, and 5.15% in the FA+PHBA treatment; and 41.40, 43.25, 4.85, and 6.55% in the CSY-P13+FA+PHBA treatment. In Proteobacteria phylum, 272 genera were detected. Among these, *Acinetobacter* was the second dominant genus, representing 1.48, 2.15, 4.05, and 3.94% in the control, CSY-P13, FA+PHBA, and CSY-P13+FA+PHBA treatments, respectively.

**FIGURE 9:**
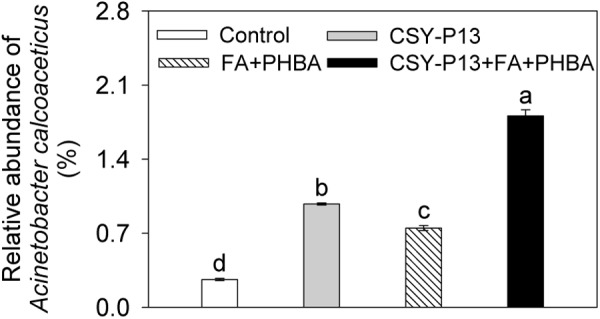
Effects of CSY-P13 and the mixture of FA and PHBA on relative abundance of one OTU, which is identified as *Acinetobacter calcoaceticus* by using sequence alignment of 16S rRNA gene sequences. Bars represent standard errors of three biological replicates. Values with the different letters are significantly different at *P* < 0.05 by LSD test using SPSS for Windows 22.0 software (IBM Corp., Armonk, NY, United States).

One kind of OTU was identified as *A. calcoaceticus* by using 16S rRNA gene sequences, and it was the first dominant species in *Acinetobacter*. Compared to control, the relative abundance of *A. calcoaceticus* was significantly (*P* < 0.01) increased in the CSY-P13 over FA+PHBA treatment (**Figure 9**). In the CSY-P13+FA+PHBA treatment in comparison to the FA+PHBA treatment, the abundance of *A. calcoaceticus* was significantly (*P* < 0.001) increased as well.

**FIGURE 10:**
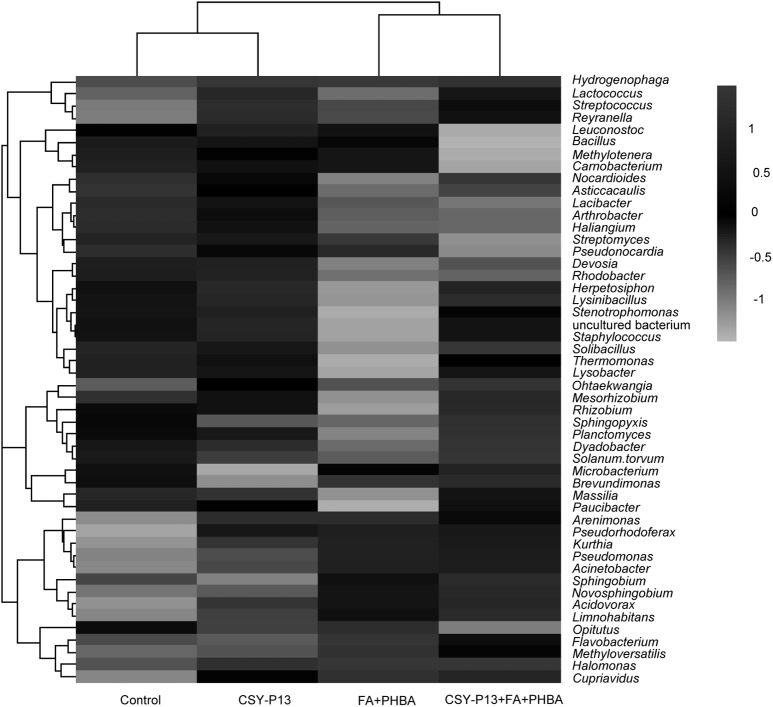
Heatmap showing the relative abundance of the top 50 abundant bacterial genera in rhizospheric soil. Phylogenetic trees are performed using the neighbor-joining method. Shading zones represent the relative abundance of different treatments within each genus. For a certain genus, when the shading intensity of one treatment is between 0 and 1, the abundance value of the genus in this treatment is higher than the mean abundance value of the genus in four treatments; when the shading intensity of one treatment is between 0 and –1, the abundance value of the genus in this treatment is lower than the mean abundance value of the genus in all treatments.

Clustering analysis was performed according to bacterial composition at the genus level, with bacteria from the same branch having higher similarity (**Figure 10**). Bacteria from the FA+PHBA and the CSY-P13+FA+PHBA treatments clustered together and were different from those from the CSY-P13 treatment and control, indicating that the mixture of FA and PHBA had a significant impact on bacterial community structure in rhizospheric soil.

A principal component analysis based on genera abundance showed distinct differences in bacterial composition of rhizospheric soil (Supplementary Figure S10). The bacterial community of the FA+PHBA treatment separated from those of control or the CSY-P13+FA+PHBA treatment by the second component (PC2, accounted for 14.1% of the variance).

## Discussion

Plant growth and MDA content are indicators of stress damage in plants (Huang et al., 2015). The mixture of FA and PHBA in this study not only decreased cucumber plant height but also increased the content of MDA in cucumber leaves, whereas CSY-P13 increased the plant height and decreased the content of MDA under conditions of FA and PHBA. There results indicated that the mixture of FA and PHBA causes stress in cucumber plants and applying CSY-P13 into cucumber-planted soil can mitigate the stress of FA and PHBA.

*Acinetobacter calcoaceticus* CSY-P13 from the cucumber rhizosphere in this study effectively degraded the mixture of FA and PHBA not only in culture solution but also in cucumber-planted soil. This can be supported by a report that *A. calcoaceticus* DSM586 have the capability to degrade benzoic acid, PHBA, and vanillic acid in culture solution (Delneri et al., 1995). We propose that CSY-P13 mitigates the stress of FA and PHBA in cucumber by degrading FA and PHBA in soil.

The metabolites 4VG, vanillin, vanillic acid, and PA were detected during FA degradation by CSY-P13 in this study. Karmakar et al. (2000) observed the same metabolites from FA degradation by *Bacillus coagulans* BK07. As shown in **Figure 1C**, we propose a pathway of FA metabolism by CSY-P13 in reference to Karmakar et al. (2000). During PHBA degradation by CSY-P13, PA was a metabolite in the current study. Similarly, PHBA is converted to PA by *Pseudomonas putida* CSY-P1 (Chen et al., 2015). The concentrations of these metabolites (4VG, vanillin, vanillic acid, and PA) all increased first and then decreased during degradation of the mixture of FA and PHBA by CSY-P13, indicating that the strain in this study can decompose the metabolites of FA and PHBA and do not result in stresses of these metabolites to plants. Similarly, *Acinetobacter* strains have the ability to degrade vanillin (Gawai et al., 2005), vanillic acid (Delneri et al., 1995), and PA (Sparnins et al., 1974).

MDA is a product of membrane lipid peroxidation, which can be induced by the overproduction of H_2_O_2_ and ${\text{O}}_{2}^{•-}$ in plants under environmental stresses (Smirnoff, 1993). Therefore, the stress caused by the mixture of FA and PHBA in the current study resulted in the enhanced levels of H_2_O_2_ and ${\text{O}}_{2}^{•-}$ in leaves, consistent with the increase in MDA content. FA-degrading *Bacillus methylotrophicus* CSY-F1 (Zhang et al., 2015) decreases the levels of H_2_O_2_, ${\text{O}}_{2}^{•-}$, and MDA in cucumber leaves under FA conditions. In this study, inoculation with CSY-P13 into cucumber-planted soil also resulted in the decreased levels of H_2_O_2_, ${\text{O}}_{2}^{•-}$, and MDA in leaves under the mixture of FA and PHBA. These results were consistent with the increased plant height in the CSY-P13+FA+PHBA treatment in comparison to the FA+PHBA treatment, indicating that CSY-P13 mitigates the stress of the two phenolic compounds by decreasing ROS levels in cucumber.

The ROS levels can be regulated by antioxidant enzymes in plants (Zhang et al., 2012), so we determined the activities of these enzymes in cucumber seedlings. As a response to the mixture of FA and PHBA in this study, the activities of SOD, CAT, APX, GSH-Px, DHAR, MDHAR, and GR were decreased in leaves. Similarly, the activity of SOD is inhibited in maize seedlings under antimony stress (Pan et al., 2011). On the other hand, inoculation by *Serratia marcescens* and *Glomus intraradices* elevates antioxidant enzyme activities in oat plantlets grown in petroleum-contaminated soil (Dong et al., 2014). When CSY-P13 has been added to cucumber-planted soil containing the mixture of FA and PHBA, the activities of SOD, CAT, APX, GSH-Px, DHAR, MDHAR, and GR were higher in leaves than those in the FA+PHBA treatment, which were consistent with the increased transcript levels of *Cu/Zn-SOD*, *Mn-SOD*, and *CAT*, in agreement with the enhanced levels of AsA and GSH, and in accord with the decreased levels of H_2_O_2_, ${\text{O}}_{2}^{•-}$, and MDA. These indicated that CSY-P13 may activate antioxidant enzymes, which play a role in decreasing ROS levels. As a result, membrane lipid peroxidation and the stress from FA and PHBA will be alleviated in cucumber.

Using high throughput sequencing, the absolute abundance of *A. calcoaceticus* in cucumber rhizosphere was higher in the CSY-P13 treatment in comparison to the control or in the CSY-P13+FA+PHBA treatment compared to the FA+PHBA treatment, suggesting that CSY-P13 colonizes roots. However, after CSY-P13 inoculation, we not only observed *A. calcoaceticus* but also obtained other bacteria in the rhizosphere. In the CSY-P13-uninoculated treatments that were regarded as the control experiments, these bacteria were observed as well. The reason may be that the soil in this study was only autoclaved and then air-dried before culturing cucumber seedlings, and bacteria besides CSY-P13 might grow. This makes it possible to investigate the effects of CSY-P13 on soil bacterial community. The dominant phyla observed in cucumber rhizosphere of the current study were Firmicutes, Proteobacteria, Actinobacteria, and Bacteroidetes. Hollister et al. (2010) also found that these four phyla were the most common dominant microflora in soil. CSY-P13 increased bacterial richness and diversity in rhizospheric soil according to the Chao1 and ACE richness estimators and the Simpson and Shannon indices, whereas the results of clustering analysis showed that the mixture of FA and PHBA had an impact on bacterial community structure as well. Similarly, applying biofertilizer containing *Bacillus amyloliquefaciens* to a banana orchard increases diversity of bacteria in soil (Shen et al., 2015), and soil phenolics change soil microbial communities (Zhou et al., 2012).

In order to further analyze the effects of CSY-P13 and the mixture of FA and PHBA on rhizospheric microorganisms, we determined the activities of soil enzymes, which reflect the composition of soil microbial community (Kim et al., 2012). Firmicutes have an important role on soil C and N cycling (Ferreira et al., 2015), and Proteobacteria and Bacteroidetes are important contributors to N cycling in soil (van Spanning et al., 2005) and induce phosphatase when inorganic phosphate is insufficient (Sebastian and Ammerman, 2009), while Actinobacteria play a critical role in soil C mineralization (Tardy et al., 2015). Therefore, the activities of urease, phosphatase, and sucrase in cucumber rhizosphere soil were determined in this study. As an indicator of aerobic microbial activity (García and Hernández, 1997), CAT activity in soil was also determined. The activities of urease, phosphatase, sucrase, and CAT were reduced by the mixture of FA and PHBA. Similarly, when exogenous PHBA was applied to cucumber-planted soil, activities of these four soil enzymes were decreased (Chen et al., 2015). On the other hand, inoculation with *S. marcescens* and *G. intraradices* to the petroleum-rich soil causes an increased activity of urease (Dong et al., 2014). In the present experiment, after CSY-P13 was inoculated to cucumber-planted soil containing the mixture of FA and PHBA, the activities of phosphatase, urease, sucrase, and CAT in rhizosphere soil were increased, consistent with decreased level of MDA and the increased level of plant height in cucumber. These results suggested that CSY-P13 affects the bacterial community in rhizospheric soil and induces soil enzymes, thus mitigating the stress of FA and PHBA in cucumber.

CAT in a phenol-degrading strain of *A. calcoaceticus* plays a role in short-term adaptation of this strain to stress induced by phenol (Benndorf et al., 2004). In CSY-P13 exposed to FA and PHBA, the activities of SOD, CAT, APX, and DHAR were elevated. We proposed that the induction of antioxidant enzymes in FA- and PHBA-degrading CSY-P13 contributes to tolerance of this strain to FA and PHBA stress.

## Conclusion

CSY-P13 from cucumber rhizosphere was identified as *A. calcoaceticus* CSY-P13. This strain effectively degraded the mixture of FA and PHBA, giving rise to 4VG, vanillin, vanillic acid, and PA. The optimum conditions for FA and PHBA degradation by CSY-P13 were 39.37°C, pH 6.97, and KH_2_PO_4_ concentration of 21.59 g L^-1^. During FA and PHBA degradation, the activities of SOD, CAT, APX, and DHAR were increased in CSY-P13. Inoculation with CSY-P13 increased plant height and decreased the levels of MDA, ${\text{O}}_{2}^{•-}$, and H_2_O_2_ in cucumber grown in soil supplemented with the mixture of FA and PHBA. Moreover, in cucumber leaves under conditions of the mixture of FA and PHBA, CSY-P13 enhanced the activities of SOD, APX, CAT, GSH-Px, DHAR, MDHAR, and GR; increased the transcript levels of *Cu/Zn-SOD*, *Mn-SOD*, and *CAT*; and elevated the contents of AsA and GSH. When CSY-P13 was inoculated into cucumber-planted soil supplemented with the mixture of FA and PHBA, the activities of phosphatase, CAT, urease, and sucrase were increased, and the bacterial richness, diversity, and community composition were affected in rhizospheric soil. Thus, *A. calcoaceticus* CSY-P13 can degrade the mixture of FA and PHBA in soil and mitigate the stress of FA and PHBA in cucumber by activating antioxidant enzymes and decreasing ROS levels, and also by changing bacterial community in rhizospheric soil and inducing soil enzymes. Antioxidant enzymes in CSY-P13 contribute to the tolerance of this strain to FA and PHBA stress during FA and PHBA degradation.

## Author Contributions

J-GB defined the research theme and designed the methods and experiments. FW wrote the manuscript. Y-QA, YA, and X-JW co-designed the experiments, discussed the analyses, and revised the paper. Z-YC, YZ, XH, C-XC, and LW co-worked on associated data collection and their interpretation.

## Conflict of Interest Statement

The authors declare that the research was conducted in the absence of any commercial or financial relationships that could be construed as a potential conflict of interest.
