# Supplementary material for: Acinetobacter calcoaceticus CSY-P13 Mitigates Stress of Ferulic and p-Hydroxybenzoic Acids in Cucumber by Affecting Antioxidant Enzyme Activity and Soil Bacterial Community
Source: Front Microbiol. 2018 Jun 14;9:1262. doi: 10.3389/fmicb.2018.01262 (PMC6010532; doi:10.3389/fmicb.2018.01262)
Supplement: Supplementary file 1 [file Table_1.PDF]

Supplementary materials

Acinetobacter calcoaceticus CSY-P13 Mitigates Stress of Ferulic and p-Hydroxybenzoic Acids in Cucumber by Affecting Antioxidant Enzyme Activity and Soil Bacterial Community

Fenghui Wu, Yan-Qiu An, Yanrong An, Xiu-Juan Wang, Zeng-Yan Cheng, Yue Zhang, Xinwei Hou, Chang-Xia Chen, Li Wang, Ji-Gang Bai*

*Correspondence: Ji-Gang Bai: baijg73@163.com; baijg@sdau.edu.cn

TABLE S1 Levels and codes of independent variables for the Box-Behnken design

Original factors	Symbol		Coded levels		
	Uncoded	Coded	-1	0	1
KH_2PO_4 concentration (g L^{-1})	X_1	x_1	5	30	55
Temperature (°C)	X_2	x_2	37	40	43
pH	X_3	x_3	6	7	8

TABLE S2 Experimental and predicted percentages of degradated FA and PHBA based on the Box-Behnken design

Run	Variable			Percentage of degraded FA (%)		Percentage of degraded PHBA (%)	
	X_1	X_2	X_3	Experimental	Predicted	Experimental	Predicted
1	-1	-1	0	54.38	55.83	58.41	60.12
2	-1	1	0	44.85	46.32	48.68	50.56
3	1	-1	0	35.91	34.44	30.64	28.75
4	1	1	0	33.56	32.11	15.93	14.22
5	0	-1	-1	44.77	44.25	53.88	55.13
6	0	1	-1	30.75	30.21	47.79	48.88
7	0	-1	1	29.29	29.83	65.31	64.23
8	0	1	1	31.50	32.02	47.64	46.38
9	-1	0	-1	44.16	43.23	59.6	56.63
10	1	0	-1	22.62	24.62	19.92	20.55
11	-1	0	1	38.11	36.12	58.33	57.70
12	1	0	1	18.20	19.13	23.11	26.08
13	0	0	0	72.16	73.14	67.80	68.27
14	0	0	0	73.09	73.14	68.96	68.27
15	0	0	0	74.28	73.14	72.19	68.27
16	0	0	0	70.21	73.14	63.58	68.27
17	0	0	0	75.98	73.14	68.80	68.27

TABLE S3 Primers for quantitative real-time PCR of antioxidant enzyme genes

Gene name	Accession number	Primer sequence (5'-3')	Product size (bp)
Cu/Zn-SOD	EF121763	F: GACTGGGCCACATTTCAACC	108
		R: GCCTTGCCATCTTCACCAA	
Mn-SOD	EF203086	F: CAATGGCGGAGGTCACATTA	195
		R: AGAGCAAGCCACACCCATC	
CAT	EF468517	F:AATGGCCGGAGGATGTGA	111
		R:CCAACGACATAGAGAAAGCCAAC	
Actin	AB010922	F: GGTCGTGACCTTACTGATGC	166
		R: CAATAGAGGAACTGCTCTTTGC	

TABLE S4 characteristics of CSY-P13

Characteristics	CSY-P13
Gram staining	-
Oxidase	-
Aerobism	+
Amylohydrolysis	-
Gelatin liquefaction	-
Nitrate deoxidization	-
Glucose fermentation	-
Catalase	+
Indole production	-
Citrate utilization	+
Glucose utilization	+
Motility	-
Sucrase	-
Mannitol utilization	-

-, negative; +, positive

TABLE S5 ANOVA for the quadratic model evaluation of the percentage of degraded FA

Source	Coefficient	Sum of squares	Mean square	F-value	Prob > F
Model		6093.00	677.00	123.95	< 0.0001**
Intercept	73.14				
X_1 (KH_2PO_4 concentration)	-8.90	633.77	633.77	116.04	< 0.0001**
X_2 (Temperature)	-2.96	70.25	70.25	12.86	0.0089*
X_3 (pH)	-3.15	79.44	79.44	14.54	0.0066*
X_1X_2	1.80	12.89	12.89	2.36	0.1684
X_1X_3	0.41	0.66	0.66	0.12	0.7384
X_2X_3	4.06	65.84	65.84	12.06	0.0104*
X_1^2	-17.14	1236.36	1236.36	226.37	< 0.0001**
X_2^2	-13.83	805.52	805.52	147.48	< 0.0001**
X_3^2	-25.23	2681.21	2681.21	490.91	< 0.0001**
Lack of fit		38.86	12.29	1.28	0.3941

R^2 = 0.9938; Adj R^2 = 0.9857; Pred R^2 = 0.9447; *, significant at the 5% level; **, significant at the 1% level

TABLE S6 ANOVA for the quadratic model evaluation of the percentage of degraded PHBA

Source	Coefficient	Sum of squares	Mean square	F-value	Prob > F
Model		5264.18	584.91	54.47	< 0.0001**
Intercept	68.27				
X_1 (KH_2PO_4 concentration)	-16.93	2292.14	2292.14	213.46	< 0.0001**
X_2 (Temperature)	-6.02	290.30	290.30	27.03	0.0013*
X_3 (pH)	1.65	21.78	21.78	2.03	0.1974
X_1X_2	-1.24	6.18	6.18	0.58	0.4729
X_1X_3	1.12	4.98	4.98	0.46	0.5177
X_2X_3	-2.90	33.60	33.60	3.13	0.1202
X_1^2	-21.63	1970.82	1970.82	183.53	< 0.0001**
X_2^2	-8.22	284.395	284.395	26.48	0.0013*
X_3^2	-6.40	172.20	172.20	16.04	0.0052*
Lack of fit		38.86	12.29	1.28	0.3941

R^2 = 0.9859; Adj R^2 = 0.9678; Pred R^2 = 0.8783; *, significant at the 5% level; **, significant at the 1% level
