# Supplementary material for: Acinetobacter calcoaceticus CSY-P13 Mitigates Stress of Ferulic and p-Hydroxybenzoic Acids in Cucumber by Affecting Antioxidant Enzyme Activity and Soil Bacterial Community
Source: Front Microbiol. 2018 Jun 14;9:1262. doi: 10.3389/fmicb.2018.01262 (PMC6010532; doi:10.3389/fmicb.2018.01262)
Supplement: Supplementary file 2 [file Image_1.pdf]

Supplementary materials

Acinetobacter calcoaceticus CSY-P13 Mitigates Stress of Ferulic and p-Hydroxybenzoic Acids in Cucumber by Affecting Antioxidant Enzyme Activity and Soil Bacterial Community

Fenghui Wu, Yan-Qiu An, Yanrong An, Xiu-Juan Wang, Zeng-Yan Cheng, Yue Zhang, Xinwei Hou, Chang-Xia Chen, Li Wang, Ji-Gang Bai*

*Correspondence: Ji-Gang Bai: baijg73@163.com; baijg@sdau.edu.cn

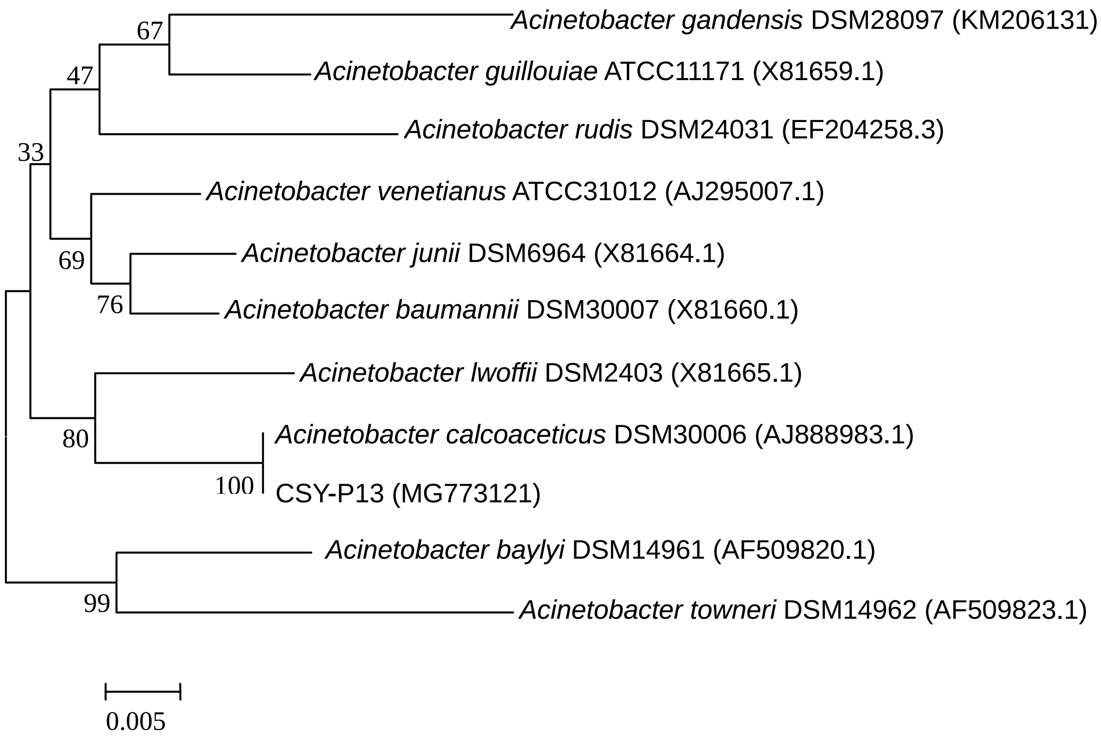

FIGURE S1 Phylogenetic tree obtained by neighbor-joining analysis of 16S rRNA gene sequences. Bootstrap values of >50% from 1000 bootstrap replicates are indicated above the branch nodes. The scale bar represents 0.02 substitutions per nucleotide position.

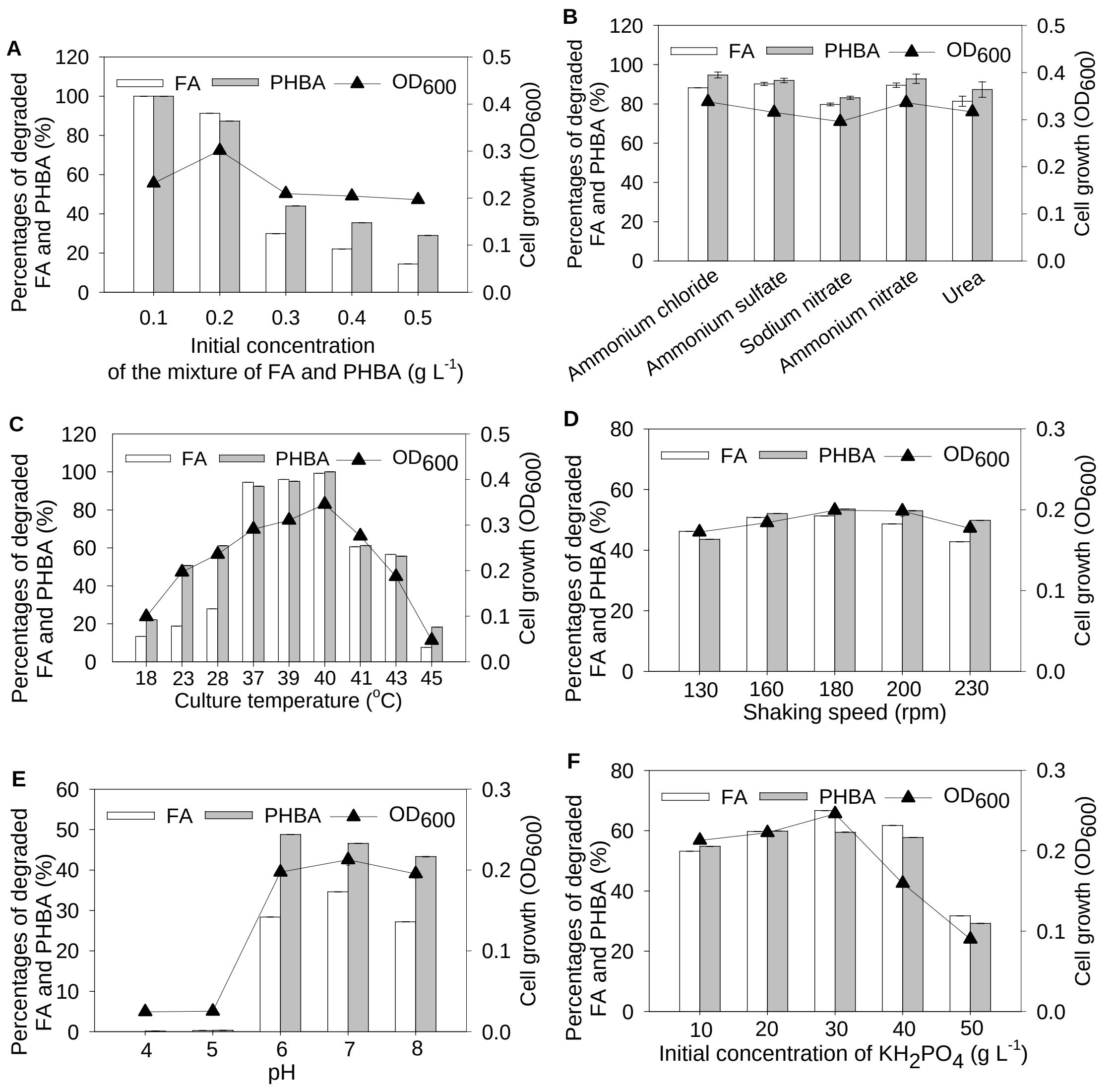

FIGURE S2 Effects of initial concentration of the mixture of FA and PHBA (**A**), nitrogen source (**B**), culture temperature (**C**), shaking speed (**D**), pH (**E**), and initial KH_2PO_4 concentration (**F**) on FA and PHBA degradation by CSY-P13.

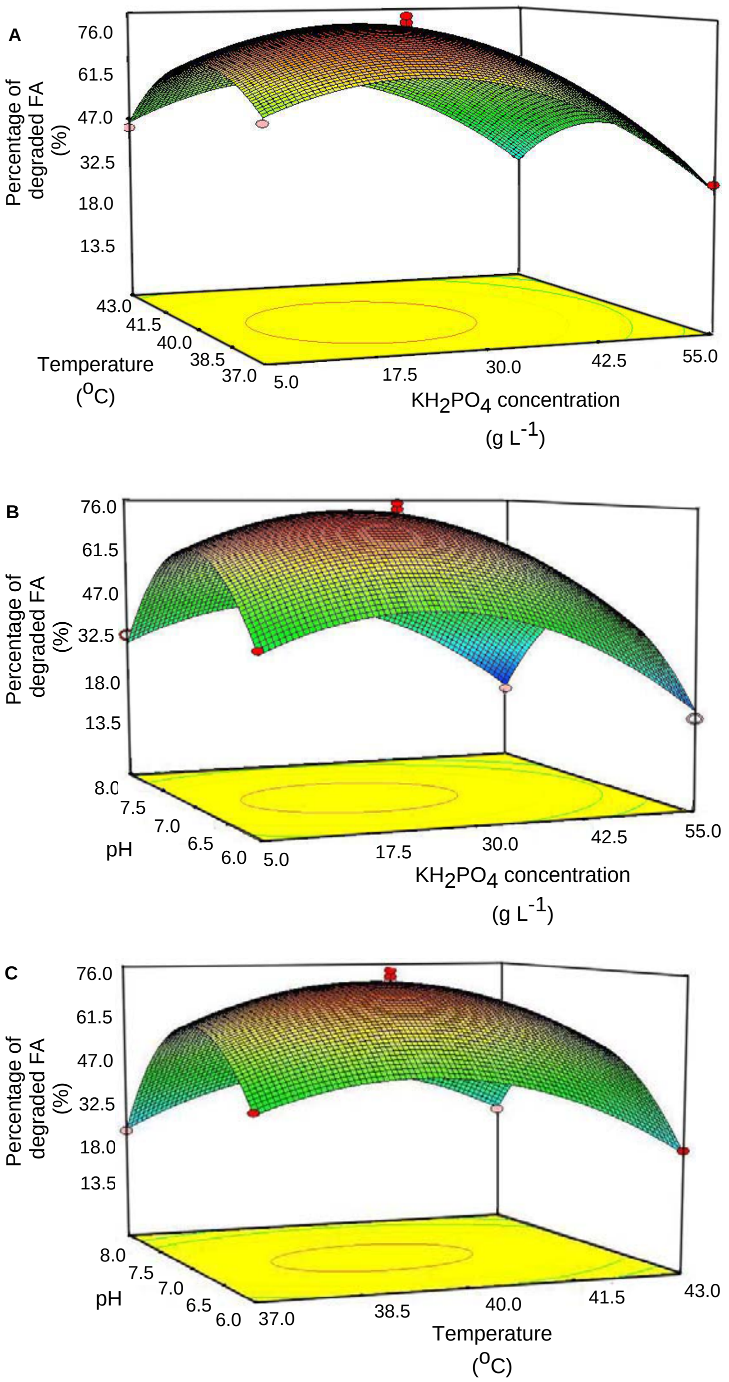

FIGURE S3 Response surface plot for the degradation of FA by CSY-P1 in terms of the effects of temperature and KH_2PO_4 concentration (**A**), pH and KH_2PO_4 concentration (**B**), and pH and temperature (**C**). Factors that were not included in the axes were fixed at their respective optimum levels.

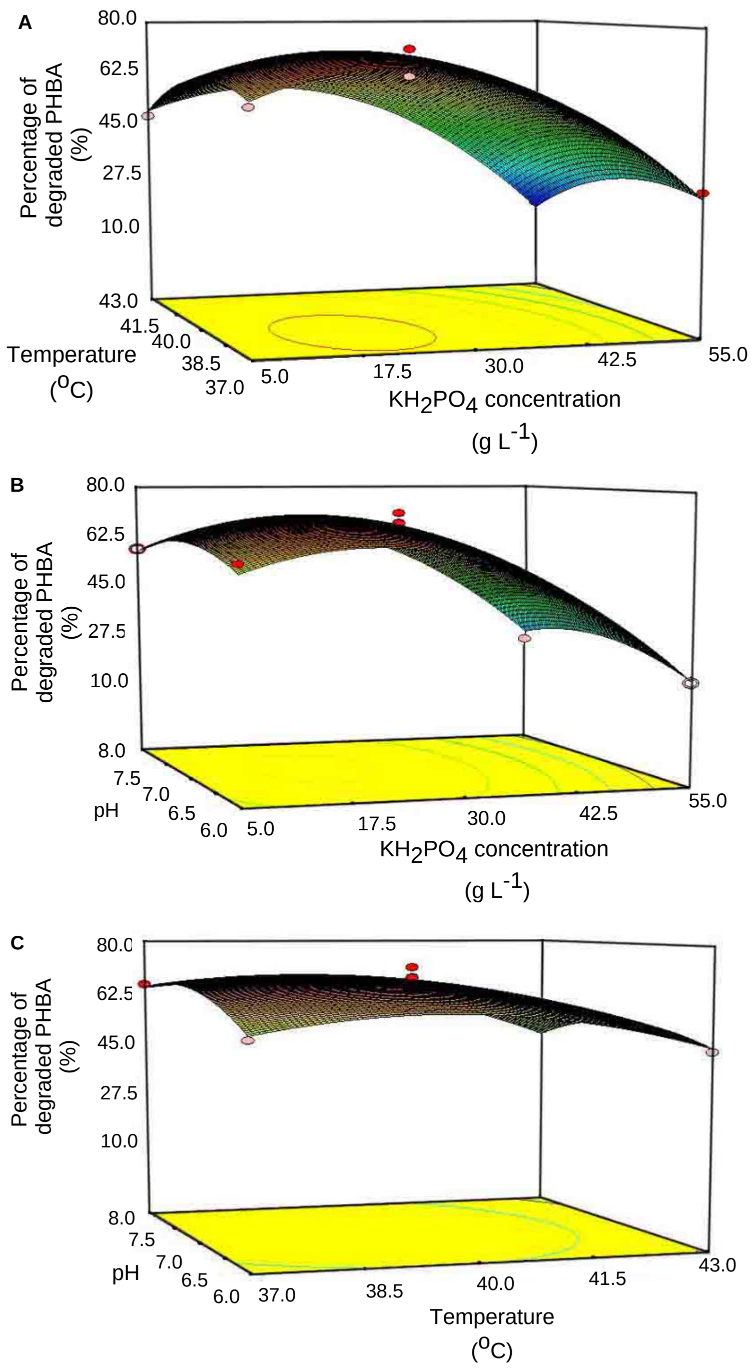

FIGURE S4 Response surface plot for the degradation of PHBA by CSY-P1 in terms of the effects of temperature and KH_2PO_4 concentration (**A**), pH and KH_2PO_4 concentration (**B**), and pH and temperature (**C**). Factors that were not included in the axes were fixed at their respective optimum levels.

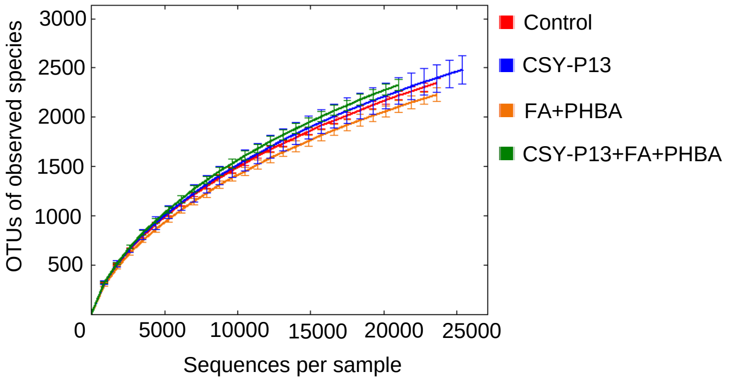

FIGURE S5 Rarefaction curves at the 97% similarity level.

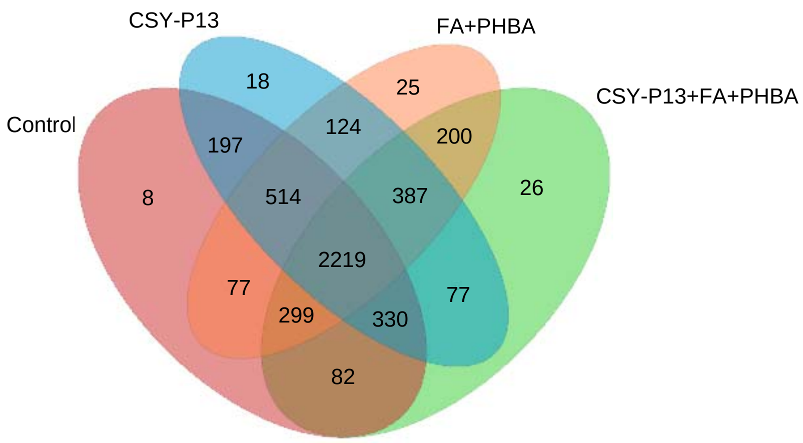

FIGURE S6 Venn diagram of bacterial richness in rhizospheric soil.

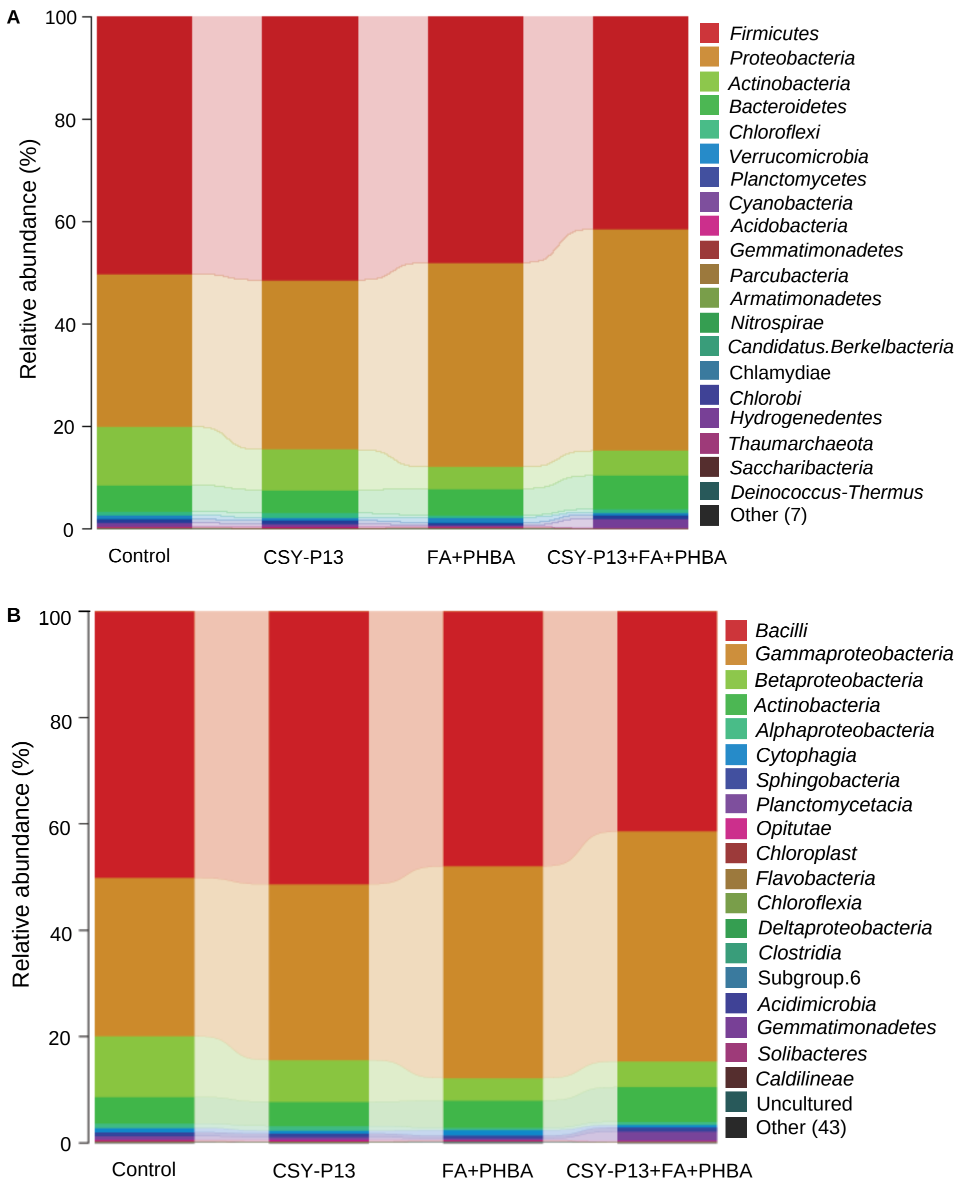

FIGURE S7 Relative abundance of bacteria phyla (**A**) and classes (**B**) in cucumber rhizospheric soil.

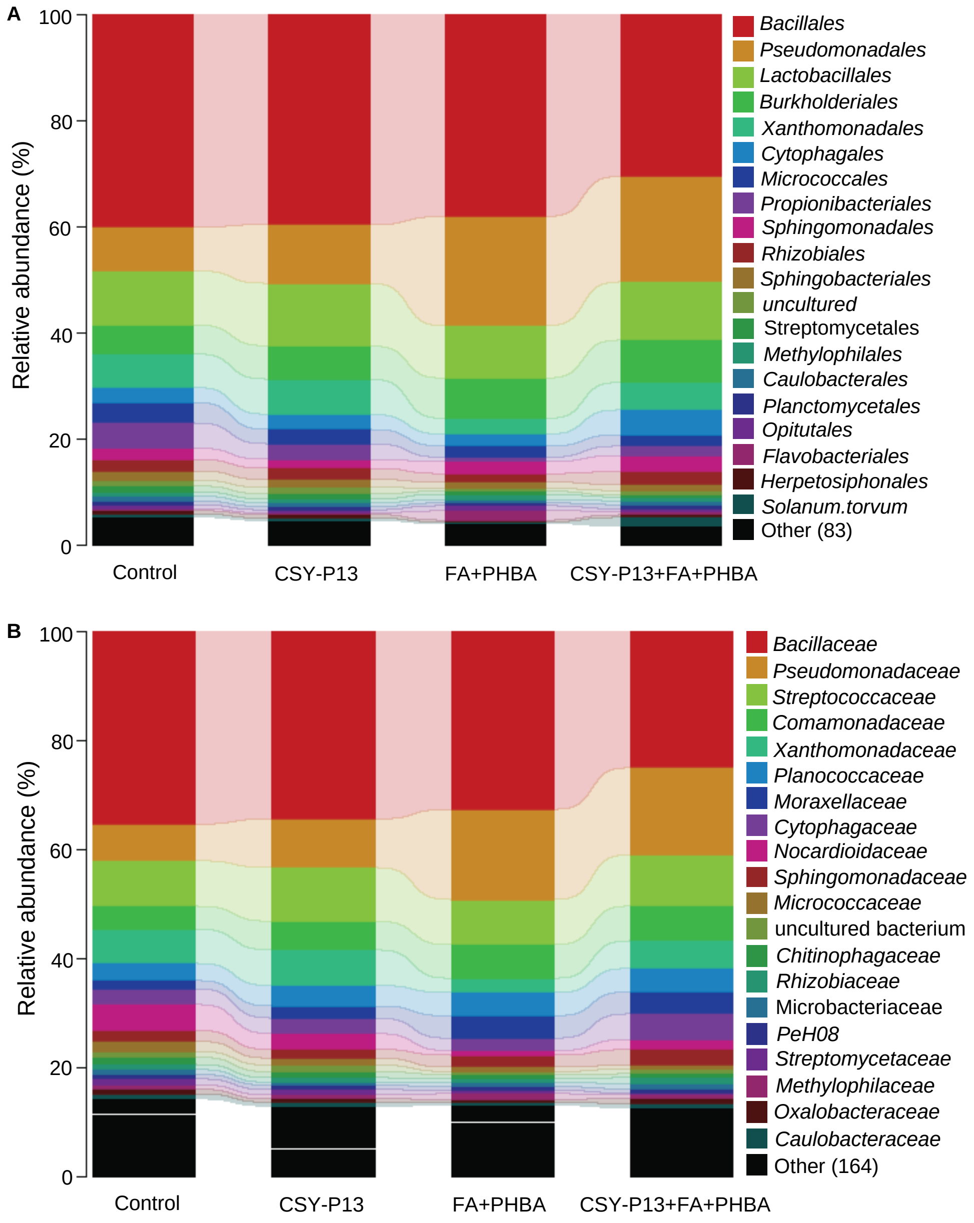

FIGURE S8 Relative abundance of bacteria orders (**A**) and families (**B**) in cucumber rhizospheric soil.

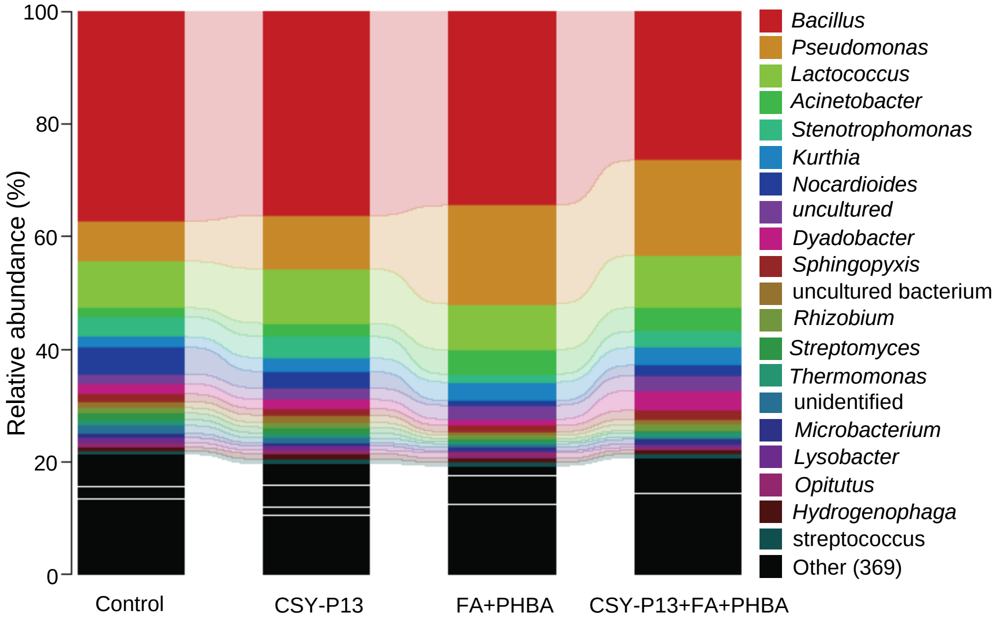

FIGURE S9 Relative abundance of bacteria genera in cucumber rhizospheric soil.

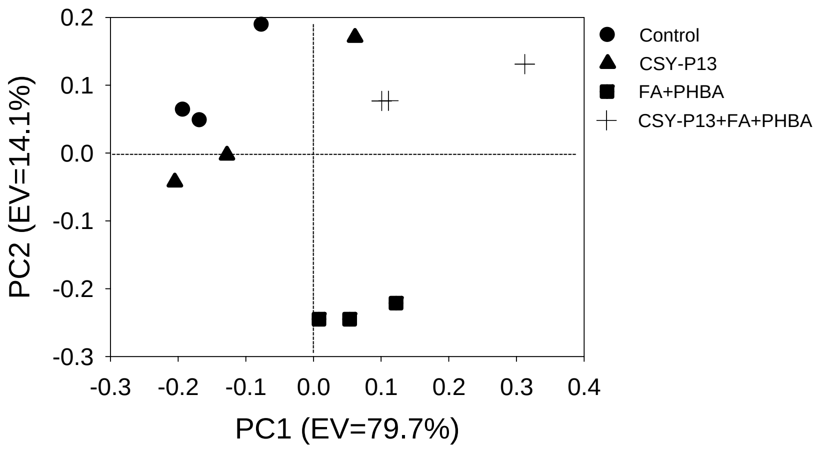

FIGURE S10 Principal component analysis performed on the relative abundance of bacteria genera in cucumber rhizospheric soil.
